# Benchmarking Foundation Models for Alzheimer’s Disease and Related Dementia Detection from Spontaneous Speech

**DOI:** 10.1093/geroni/igaf122.4077

**Published:** 2025-12-31

**Authors:** Jingyu Li, Lingchao Mao, Hairong Wang, Zhendong Wang, Xi Mao, Xuelei Ni

**Affiliations:** Georgia Institute of Technology, Atlanta, Georgia, United States; Georgia Institute of Technology, Atlanta, Georgia, United States; The University of Texas at Austin, Austin, Texas, United States; Shandong Mental Health Center, Jinan, Shandong, China (People’s Republic); University of Texas Rio Grande Valley, Brownsville, Texas, United States; Kennesaw State University, Kennesaw, Georgia, United States

## Abstract

Alzheimer’s disease and related dementias (ADRD) are progressive neurodegenerative conditions where early detection is critical for timely intervention and care planning. Acoustic biomarkers—such as changes in prosody, fluency, and pause patterns—can be extracted from spontaneous speech and offer a non-invasive avenue for early diagnosis. Foundational speech and language models, which are pre-trained deep learning models, can generate high-dimensional embeddings that capture rich contextual and acoustic information from raw audio or text. Using data from the PREPARE Phase 2 Challenge, which includes recordings from over 1,600 individuals, we examined the potential of foundation models for ADRD detection. Specifically, we benchmarked a range of open-source speech and language models on their ability to classify participants into different stages of cognitive decline. Among speech-based approaches, the Whisper-medium model achieved the highest performance (accuracy = 0.731; AUC = 0.802). Overall, state-of-the-art automatic speech recognition (ASR) models outperformed other models. Incorporating prosodic features such as pauses also improved classification in text-based approaches. Our work presents a comprehensive benchmarking framework built on state-of-the-art speech models and validated on a large, clinically relevant dataset, and demonstrates that foundation models can provide a scalable and cost-effective framework for early ADRD detection. Our findings highlight the promise of speech-derived embeddings as powerful non-invasive biomarkers for cognitive impairment. Future ADRD screening approaches should integrate both semantic and non-semantic features to improve generalizability and support broader clinical application.

